# Editorial: Neurobiology and Cognition Across the Autism-Psychosis Spectrum

**DOI:** 10.3389/fpsyt.2021.654246

**Published:** 2021-02-10

**Authors:** Noah J. Sasson, Amy E. Pinkham, Tim B. Ziermans

**Affiliations:** ^1^Department of Psychology, School of Behavioral and Brain Sciences, The University of Texas at Dallas, Richardson, TX, United States; ^2^Department of Psychology, Social and Behavioral Sciences, University of Amsterdam, Amsterdam, Netherlands

**Keywords:** autism, schizophenia, psychosis, social cognition, neurocognition, neurobiology

Although Autism Spectrum Conditions (ASC) and Schizophrenia Spectrum Conditions (SSC) are recognized as distinct diagnostic categories with independent features, they share a history of clinical entanglement stemming from overlapping symptomatology, particularly in the area of social behavior (1). Examination of cognition and the neurobiology of ASC and SSC, both in the conditions themselves and within their subclinical manifestations, offers potential for illuminating the shared and unique mechanisms underlying their social characteristics. In recent years, both direct comparisons of ASC and SSC and continuous explorations of the autism-psychosis spectrum have begun to show promise for producing more precise segmentation and greater clinical utility than traditional comparisons to non-clinical controls. Such studies ultimately may help to inform etiological understanding, improve screening and diagnosis, and provide more targeted support.

The goal of this Frontiers Research Topic is to showcase innovative new work examining cognitive and neurobiological features of the autism-psychosis spectrum. Consistent with the primary focus of research activity in this area, included articles can be organized under two primary subsections: Cognition and Neurobiology.

Collectively, the articles on cognition suggest that both ASC and SSC are often (but not always) characterized by neuro- and social cognitive differences compared to non-affected controls that relate in both direct and indirect ways to the broader functional and social disabilities associated with the conditions. For instance, Sijtsma et al. demonstrate that adolescents with more autistic-like experiences are less likely to be nominated as friends by their peers despite no relation found between autistic-like experiences and social cognitive performance or self-reported rates of friendships. This suggests that traditional social cognitive measures may not always capture the social differences associated with subclinical autistic characteristics affecting peer relationships. Relatedly, Larson et al. found that autistic people who have experienced psychosis had higher rates of schizotypy and emotional difficulties than both neurotypical controls and autistic people with no psychosis history despite little to no difference between the groups on measures of perspective taking. Their findings also highlight a previously under-recognized clinical characteristic associated with schizotypal traits: high affective lability, which refers to elevated shifting between different emotional states. Likewise, the work by van der Linden et al. also suggests that the full impact of psychotic experiences (PE) among autistic individuals are not well-understood. Their paper indicates that autistic people do not differ from controls in lifetime PE but do report more frequent momentary PE and greater distress associated with their PE. They conclude that stress may serve as an important risk factor for PE among autistic individuals. Meanwhile, the papers by Maat et al. and Abu-Akel et al. demonstrate that the presence of cross-diagnostic symptoms along the autism-psychosis spectrum may produce novel cognitive effects in autistic people not seen in autism alone. In Maat et al., response time latency to social and non-social stimuli was increased among autistic adolescents who present with features of psychosis. Specifically, those with attenuated psychosis syndrome, a condition defined by subclinical positive symptoms associated with risk of subsequent psychosis, did not differ from controls on measures of pattern, face, or emotion recognition, but exhibited slower response times to stimuli. Similarly, Abu-Akel et al. found that autistic people with co-occurring schizotypal personality disorder (SPD) and higher positive psychotic symptoms exhibited more sustained (but not inhibitory) attention relative to individuals with autism or SPD alone. Such findings suggest that autistic and positive symptoms may diametrically influence sustained attention and, more broadly, highlight the need for better understanding the relationship between autism and SPD and the effects of dual diagnosis.

The paper by Deste et al. also reports combinatory effects, but unlike the Larson et al. and Maat et al. papers that examined autistic people with features of psychosis, their study focuses on autistic symptoms among patients with schizophrenia and their association with relationship outcomes. In line with a recent large-scale study in psychotic patients and their siblings (2), they find that higher autism symptoms constitute a significant predictor of poor social relationships in schizophrenia, even after controlling for other relevant demographic and clinical variables. Autism and schizophrenia, however, are not only characterized by social difficulties but also multisensory processing difficulties, and the paper by Noel et al. offers a direct comparison of visual-tactile spatial multisensory processing in the two conditions. They find that both groups do not differ from non-affected controls on a cross-modal congruency, but autistic adults exhibit a smaller and more restricted peri-personal space (i.e., the space immediately around the body) than both controls and adults with schizophrenia. Additionally, they find an association between smaller peri-personal space and social symptoms, suggesting a link between some aspects of multisensory processing and social-emotional functioning. Next, Kuo and Eack provide the first systematic review and meta-analytic comparison of non-social cognitive abilities in autism and schizophrenia. In contrast to studies of social cognition that do not show many performance differences between the groups (3–5), their comparison revealed superior performance in autism on working memory, visuospatial memory and learning, language, and comparable performance on processing speed, attention, and verbal comprehension. These distinguishable cognitive profiles extended from adolescence to middle adulthood and collectively indicate important neurocognitive differences that may serve as distinguishing mechanisms of their overlapping reductions in social cognitive performance.

As many of these studies highlight, clinical differentiation across the autism-psychosis spectrum can be difficult among those presenting with co-occurring mental health conditions and shared social characteristics. One paper in the collection (Demetriou et al.) examines the utility of using machine learning to differentiate autism, early psychosis, and social anxiety disorder based on a comprehensive battery of neurocognitive, social cognitive, and mood assessments. Social cognition, visuospatial memory, and mood (e.g., depression, anxiety, and stress) differentiated the clinical groups from a neurotypical control group and the social anxiety group from the autism and early psychosis groups. The autism and early psychosis groups were more difficult to differentiate, with only psychomotor speed and stress distinguishing them.

The cognitive differences characterizing the autism-psychosis spectrum are supported by an underlying neurobiology that five papers in this collection seek to better understand. First, Barlati et al. offer an incisive review of the shared and divergent neuroanatomical, neurofunctional, and molecular markers of social cognition in autism and schizophrenia. Consistent with the goals of this Frontiers Research Topic, their review is organized in accordance with a Research Domain Criteria perspective that seeks to identify behavioral and biological signatures of clinical symptoms spanning diagnostic conditions, and as such serves as a useful summary and guide for future research initiatives. Relatedly, Nair et al. provide a comprehensive narrative review of functional connectivity studies of social cognition in autistic adolescents and those with early-onset psychosis and contextualize these findings within a developmental framework. They conclude that disruptions in default mode network connectivity are associated with social difficulties in both groups but are less relevant to other features of each condition. Meanwhile, Samaey et al. provide a more focused review of the neural underpinnings of one social cognitive ability in particular, facial expression processing, and conclude that altered connectivity and activation in the fusiform gyrus and amygdala among autistic individuals and those with primary psychosis may be influenced by adverse childhood events. They argue that more integrative studies across clinical conditions, framed within a developmental context, are needed to fulfill the promise of identifying selective biomarkers.

Conclusions from these three reviews are supported by two empirical papers in the collection. Brady et al. examined associations between brain connectivity and social cognition in sample of people with psychosis and neurotypical controls and found evidence across both samples that implicated a cerebellar-parietal circuit strongly linked with social cognitive ability. They highlight this circuit as a potential trans-diagnostic biomarker of reduced social cognitive performance. Meanwhile, Foss-Feig et al. leveraged a longitudinal sample to examine the P300 ERP component as a potential indicator of conversion to psychosis among individuals at clinical high risk (CHR) with a history of autism. Unlike previous findings suggesting that P300 amplitude reductions predict psychosis in CHR populations without autism, they found enhanced neural responses during attentional orienting tasks in their small sample of individuals with combined CHR and autism. Such a result is consistent with other papers in this Research Topic indicating that the combination of symptoms across the autism-psychosis spectrum may interact in unpredicted ways and not follow a simple main effect framework.

## Author Contributions

NS wrote the first draft of the manuscript. AP and TZ contributed intellectually to the manuscript and helped revise it. All authors have read and approved the submitted version.

## Conflict of Interest

The authors declare that the research was conducted in the absence of any commercial or financial relationships that could be construed as a potential conflict of interest.
